# Complete Genome Sequence Analysis of *Kribbella* sp. CA-293567 and Identification of the Kribbellichelins A & B and Sandramycin Biosynthetic Gene Clusters

**DOI:** 10.3390/microorganisms11020265

**Published:** 2023-01-19

**Authors:** Marina Sánchez-Hidalgo, María Jesús García, Ignacio González, Daniel Oves-Costales, Olga Genilloud

**Affiliations:** Fundación MEDINA, Avenida del Conocimiento 34, PTS Health Sciences Technology Park, 18016 Granada, Spain

**Keywords:** *Kribbella*, sandramycin, kribbellichelin, genome mining, biosynthetic gene cluster, minor genera of actinomycetes

## Abstract

Minor genera actinomycetes are considered a promising source of new secondary metabolites. The strain *Kribbella* sp. CA-293567 produces sandramycin and kribbellichelins A & B In this work, we describe the complete genome sequencing of this strain and the in silico identification of biosynthetic gene clusters (BGCs), focusing on the pathways encoding sandramycin and kribbellichelins A–B. We also present a comparative analysis of the biosynthetic potential of 38 publicly available genomes from *Kribbella* strains.

## 1. Introduction

The increasing number of antibiotic resistant strains has boosted the search of new antibiotics that are urgently needed against pathogenic bacteria [1,2,3,4]. Natural products represent the most important source of novel antibiotics, being the actinomycetes the most prolific producers of natural bioactive compounds [5,6,7,8]. Actinomycetes are Gram-positive filamentous bacteria with a high G+C DNA content [9,10]. More than 50% of the bioactive compounds produced by actinomycetes have been isolated from the predominant genus *Streptomyces* [9,11]. However, in recent decades, minor genera of actinomycetes, also known as rare actinomycetes, have received considerable research attention [8,12]. Minor genera of actinomycetes have been found in a wide variety of underexplored terrestrial and aquatic ecosystems [13,14,15], including soil, sediments, stones, mangroves, plants, lichens and animals from a broad range of environments [16]. From the 220 minor genera of actinomycetes described so far, some of them can be more frequently isolated from different sources and have been shown to produce a high diversity of secondary metabolites: *Actinocorallia*, *Actinomadura*, *Actinoplanes*, *Amycolatopsis*, *Kibdelosporangium*, *Kribbella*, *Micromonospora*, *Nocardia*, *Nocardiopsis*, *Nonomuraea*, *Pseudonocardia*, *Rhodococcus*, *Saccharopolyspora* or *Streptosporangium* [14,16]. Some of these genera are well known as producers of relevant antibiotics with clinical applications, including rifamycins from *Amycolatopsis mediterranei*, vancomycin from *Amycolatopsis orientalis*, erythromycin from *Saccharopolyspora erythraea*, teicoplanin from *Actinoplanes teichomyceticus*, and gentamicin from *Micromonospora purpurea* [17]. The importance of minor genera of actinomycetes has been corroborated by the discovery of several novel compounds in the last years [14,15,16,17,18,19,20,21,22,23], such as kongjuemycins [23], catellatolactams [24], and persicamidines [25], so these genera represent an untapped resource of potential new bioactive natural products [17,26].

The significant advances in bacterial genome sequencing and the development of bioinformatic genome mining tools, has resulted in the identification of an extraordinary high number of silent or cryptic secondary metabolite BGCs encoding potentially novel compounds that have yet to be identified and are not expressed under standard laboratory culture conditions [27,28,29,30,31,32,33]. The growing number of reports on the discovery of cryptic gene clusters from sequenced minor genera of actinomycetes highlights increasing opportunities to discover new bioactive natural products from this broad diversity of lineages [34].

**Figure 1:**
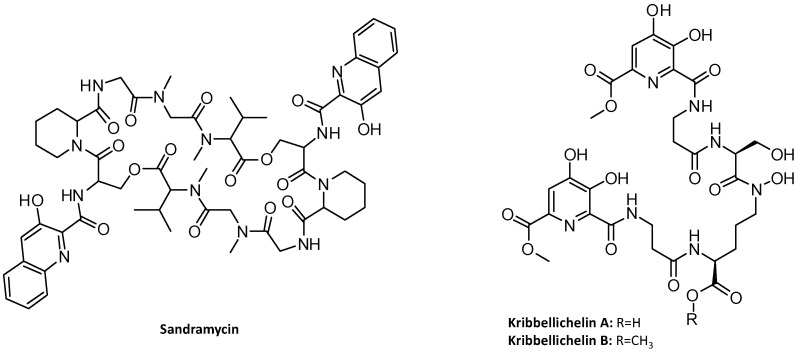
Structures of sandramycin and kribbellichelins A and B.

The genus *Kribbella* belongs to the family *Kribbellaceae* (order *Propionibacteriales*) and currently consists of 33 species [35,36]. Some active secondary metabolites have been described from *Kribbella* strains: the kribellosides A–D, RNA 5′-triphosphatase inhibitors from *Kribbella* sp. MI481-42F6 [37]; the cyclic decadepsipeptide antitumor antibiotic sandramycin (Figure 1) from *K. sandramycini* ATCC 39419^T^ [38,39] and the antibiotics kribbellichelins A-B from the recently described *Kribbella* sp. CA-293567 (Figure 1) [40]. The strain *Kribbella* sp. CA-293567 has also been shown to produce sandramycin, which additionally exhibits antifungal activity [40].

There are currently 38 sequenced genomes of *Kribbella* strains in NCBI, including the genome of the sandramycin producer *K. sandramycini* ATCC 39419^T^. Sandramycin was described nearly 30 years ago, but its BGC was only mentioned in a recent publication describing the biosynthetic gene cluster of luzopeptins and korkomicins [41]. However, a detailed analysis of the genes involved in sandramycin biosynthesis has not been reported yet.

In this work, we present the complete genome sequence analysis of the strain *Kribbella* sp. CA-293567 and the identification of the putative BGCs present in the genome, focusing on the BGCs encoding kribbellichelins and sandramycin. The comparative genome analysis of all publicly available sequenced *Kribbella* strains has permitted us to perform a detailed analysis of the biosynthetic potential among the species of the genus.

## 2. Materials and Methods

### 2.1. Strains

The strain *Kribbella* sp. CA-293567 was isolated from the endemic plant *Limonium majus*, collected in El Saladar del Margen, in Cúllar-Baza depression, Granada (Spain). The strain showed a 99.41% sequence similarity to the strain *Kribbella koreensis* LM 161^T^ (GenBank Accession No. Y09159), according to the 16S rRNA gene sequence analysis [40].

### 2.2. DNA Extraction

High molecular weight genomic DNA was isolated following the protocol described in [42] from 10 mL bacterial culture grown on ATCC-2 liquid medium (soluble starch 20 g/L, glucose 10 g/L, NZ Amine Type E 5 g/L, meat extract 3 g/L, peptone 5 g/L, yeast extract 5 g/L, calcium carbonate 1 g/L, pH 7) grown on an orbital shaker at 28 °C, 220 rpm, 70% relative humidity for 4 days. 

### 2.3. Whole Genome Sequencing

The genome of the strain CA-293567 was sequenced, de novo assembled and annotated by Macrogen (Seoul, Republic of Korea; http://www.macrogen.com, accessed on 27 April 2020), using a combined strategy of Illumina Novaseq 6000 and PacBio RSII platforms [43,44]. The PacBio long-reads were assembled with Microbial Assembly Application (https://www.pacb.com/wp-content/uploads/SMRT-Link-User-Guide-v8.0.pdf, accessed on 27 April 2020), and then Illumina reads were mapped to the assembly for accurate genome sequence and error correction using Pilon (v1.21) [45]. After mapping, the consensus sequence was generated. The completeness of the genome was assessed with BUSCO (Benchmarking Universal Single-Copy Orthologous, v5.1.3) [46].

### 2.4. Bioinformatic Analysis

The genomic sequence of strain CA-293567, as well as the publicly available genomes of other *Kribbella* strains (Table S1) were analyzed with antiSMASH v6.1.1 [47] and PRISM v4 [48] to identify putative secondary metabolites BGCs. BLAST (Basic Local Alignment Search Tool) [49] was also employed to predict the function of the genes. BiG-SCAPE (Biosynthetic Gene Similarity Clustering and Prospecting Engine) and CORASON (CORe Analysis of Syntenic Orthologs to prioritize Natural Product Biosynthetic Gene Clusters) algorithms [50] were used to generate sequence similarity networks, that were visualized using Cytoscape (v3.9.1) [51]. Genome annotation and visualization were performed in the Department of Energy Systems Biology Knowledgebase (KBase, http://kbase.us/, accessed on 19 October 2020) [52] using Prokka v1.14.5 [53] and the Circular Genome Visualization Tool (CGViewAdvanced v0.0.2) [54], respectively.

## 3. Results and Discussion

### 3.1. Whole Genome Sequencing

*Kribbella* sp. CA-293567 genome sequence was obtained with a combination of de novo PacBio and Illumina approaches yielding one circular contig of 7,611,196 bp, with a G+C DNA content of 68.6%. The genome was 100% complete according to the BUSCO analysis results. No extrachromosal elements were identified (Table S2).

**Figure 2:**
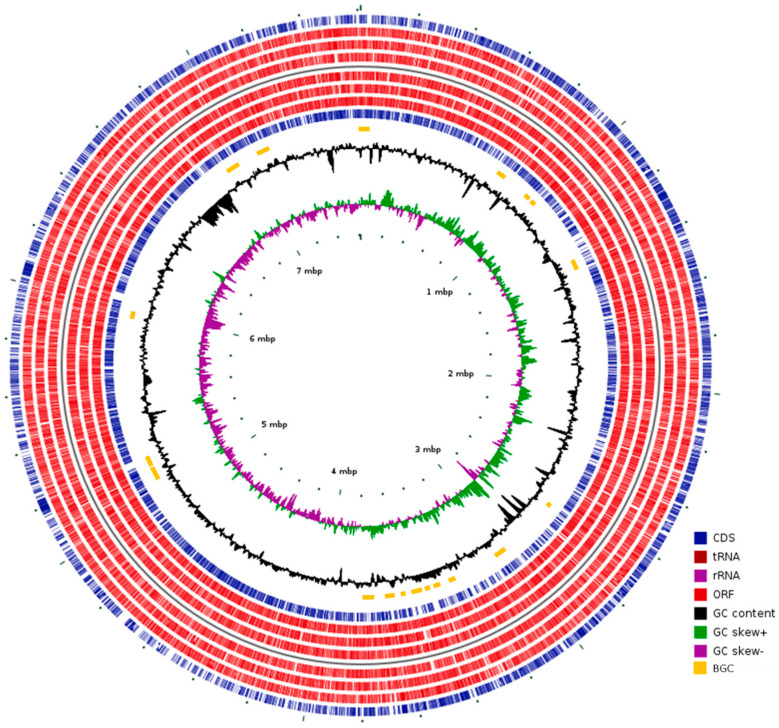
Graphical circular map of the chromosome of *Kribbella* sp. CA-293567 showing, from inner to outer layers, GC skew, GC content, location of BGCs, ORFs and CDSs in the reverse strand, and ORFs and CDSs in the forward strand.

The genome was analyzed with Prokka and a total of 7,057 genes were identified. Among them, 6982 were identified as protein-coding genes (CDSs) and their functions were assigned (Table S2, Figure 2). Only 45.80% of the CDSs (45.30% of the total genes) were assigned with a putative function while those remaining were annotated as hypothetical protein CDS, thus highlighting the need to deepen the study of the functions of these genes.

The complete genome sequence has been deposited in GenBank under the reference [CP114065].

### 3.2. Secondary Metabolites BGC Analysis

The complete genome sequence was analyzed with antiSMASH and PRISM and a total of 19 regions putatively encoding BGCs were identified, including three NRPS, three PKS, two terpenes, two RiPPs and three lanthipeptides (Table 1, Figure 2). Interestingly, only two of the predicted BGCs showed 100% similarity with the known BGCs geosmin [55] and alkylresorcinol [56]; two showed a 60% similarity with the catenulipeptin BGC [57] and the rest showed <60% similarity with known BGCs, indicating that they could synthesize new molecules. The BGCs were found to be distributed throughout the genome (Figure 2). The total length of the predicted BGCs is about 653 Kb, comprising the ~8.6% of the *Kribbella* sp. CA-293567 genome. The number of predicted BGC in relation to the genome size is among the average proportion found in prokaryotes (2.4 clusters per Mb) [58], although it is much lower than other prolific genera such as *Streptomyces*, which can reach 22% of its genome devoted to the biosynthesis of secondary metabolites [58].

### 3.3. Identification of the Kribbellichelins A and B BGC

**Figure 3:**
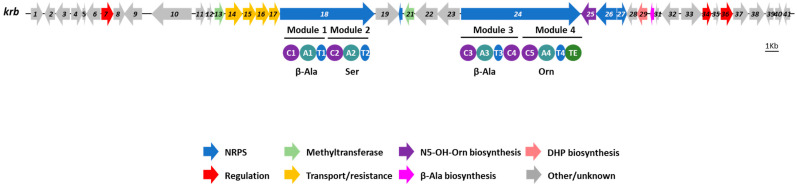
Biosynthetic gene cluster of kribbellichelins A–B (*krb*). The modules and domains present in the NRPS genes (*krb18* and *krb24*) are depicted. The amino acids activated by each adenylation domain are indicated. (A): adenylation domain; (C): condensation domain; (T): thiolation domain; (TE): thioesterase domain.

Among the BGCs identified in the genome by antiSMASH, the region 13 putatively contains a NRPS BGC with a 25% similarity with the amychelin BGC (Table 1, Figure 3). This is the only NRPS BGC of the genome containing four modules for which the analysis of the substrate specificity of the adenylation domains suggest incorporation of β-Ala, Ser, β-Ala and Orn (Figure 3), thus in good agreement with the structures of kribbellichelins A and B, which contain β-Ala, N5-OH-Orn, Ser and β-Ala (Figure 1). 

The BGC proposed for kribbellichelins A & B BGC (*krb*) spans ~59 Kb and contains 41 Open Reading Frames (ORFs) (Table S3). In addition to the NRPS genes, the BLAST analysis of the proteins encoded by these ORFs shows the presence of an aspartate 1-decarboxylase (Krb30) that could be involved in the biosynthesis of β-Ala from Asp [59]; enzymes related with the biosynthesis of dipicolinic acid [60], transcriptional regulators and transporters.

**Figure 4:**
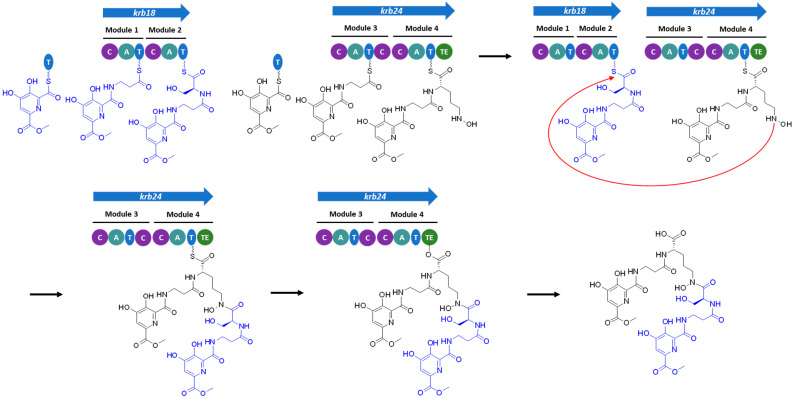
Proposed non-canonical assembly line of kribbellichelins A–B. Two dipeptides are independently synthesized by the two NRPSs, followed by amide bond formation between the nitrogen at the δ carbon of N5-OH-Orn and the carboxylic group of Ser.

Kribbellichelins A and B also harbor two moieties of methyl 6-carbonyl-4,5-dihydroxypicolinate connected to carbons C-11 and C-27 of the β-Ala units [40]. According to PRISM, an AMP-binding protein present in the cluster (Krb26) is predicted to activate 3-hydroxypicolinic acid, as it has been described in the case of SanJ, an ATP-dependent picolinate CoA ligase involved in nikkomycin biosynthesis [61]. SanJ and Krb26 share a 35.8% identity and 49.8% similarity. Thus, Krb26 could be involved in the activation of the methyl 6-carbonyl-4,5-dihydroxypicolinate moieties, that would be loaded onto a thiolation (T) domain encoded elsewhere in the genome (Figure 4). A gene related with the meso-diaminopimelate pathway (lysine biosynthesis), *krb29*, is present in the cluster. BLAST analysis of Krb29 showed that it is a 4-hydroxytetrahydrodipicolinate reductase homologous to DapB (90% identity, 95% similarity, Table S2), which catalyzes the reduction of (2*S*,4*S*)-4-hydroxy-2,3,4,5-tetrahydrodipicolinate to 2,3,4,5-tetrahydrodipicolinate in an NADH/NADPH dependent reaction [60,62]. The role of Krb29 in the biosynthesis of the methyl 6-carbonyl-4,5-dihydroxypicolinate moieties present in kribbellichelins would require further investigation. It is probable that any of the oxidases and methyltransferases present in the cluster may hydroxylate and methylate dipicolinic acid to yield methyl 6-carbonyl-4,5-dihydroxypicolinate.

Both NRPSs (Krb18 and Krb24) contain two modules including the expected condensation (C), adenylation (A), and thiolation (T) domains. The substrate specificity analysis of the A domains performed with antiSMASH and PRISM suggest incorporation of β-Ala, l-ser, β-Ala and Orn for modules 1–4, respectively (Figure 3). The thioesterase (TE) domain present in the second NRPS (Krb24) would hydrolyze the peptidic chain. An additional condensation domain is present between the two modules present in Krb24 (Figure 3). 

According to the predictions described above and to the co-linearity rule [63], the peptide sequence of kribbellichelins A and B would be β-Ala-Ser-β-Ala-N5-OH-Orn. However, the order of the amino acids in the structure is β-Ala-Ser-N5-OH-Orn-β-Ala, and two moieties of methyl 6-carbonyl-4,5-dihydroxypicolinate are connected to the β-Ala units. Thus, we propose a non-canonical assembly of kribbellichelins A and B (Figure 4): the activated methyl 6-carbonyl-4,5-dihydroxypicolinate would be transferred to the β-amino groups of the β-Ala amino acids loaded into the T domains of modules 1 and 3 of Krb18 and Krb24, respectively. This would then be followed by the independent assembly of methyl 6-carbonyl-4,5-dihydroxypicolinate-β-Ala-Ser by Krb18 and methyl 6-carbonyl-4,5-dihydroxypicolinate-β-Ala-N5-OH-Orn by Krb24. Finally, amide formation between the δ carbon of N5-OH-Orn and the carboxylic group of Ser (Figure 4), which might be catalyzed by the extra condensation domain found in Krb24, would lead to kribbellichelin A. One of the methyltransferases present in the cluster would methylate kribbellichelin A at carbonyl C-23 to yield kribbellichelin B [40]. A similar post-assembly connection of two separate chains synthesized in parallel assembly lines has been proposed for the biosynthesis of the linear polyketide alpiniamide A by a hybrid PKS-NRPS [64]. Further experiments will be required to confirm this type of non-linear NRPS assembly.

### 3.4. Identification of the Sandramycin BGC

In the course of this work, the cluster responsible for the biosynthesis of sandramycin (*sdm*) was annotated in the genome of *Kribbella sandramycini* ATCC 39419^T^ [41]. However, a detailed analysis of the genes involved in sandramycin biosynthesis has not been reported so far. 

**Figure 5:**
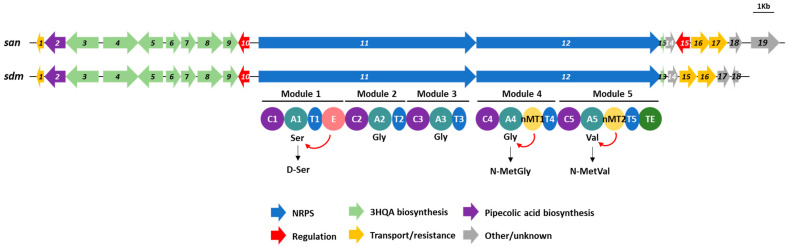
Biosynthetic gene cluster of sandramycin from *Kribbella* sp. CA-293567 (*san*) and *Kribbella sandramycini* ATCC 39419^T^ (*sdm*). The modules and domains present in the NRPS genes (*san11* and *san12*) are depicted. The amino acids proposed to be activated by each adenylation domain and the modifications performed by the E and nMT domains are indicated. (A): adenylation domain; (C): condensation domain; (E): epimerization domain; (nMT): *N*-methyltransferase domain; (T): thiolation domain; (TE): thioesterase domain.

Based on the antiSMASH results and on the sandramycin structural characteristics [39], the putative sandramycin BGC, showing a 34% similarity to that of thiocoraline [65], was identified in the genome of the strain CA-293567. This BGC (*san*) has nearly the same organization of the *sdm* cluster, containing 18 ORFs and spanning 37 Kb (Figure 5, Table S4).

Pipecolic acid, present in sandramycin [39], is a non-proteinogenic amino acid widely distributed in microorganisms, plants and animals, and is an important precursor of many natural products, such as meridamycin [66] or rapamycin [67]. The lysine cyclodeaminase RapL has been identified as the enzyme responsible for the generation of the pipecolate moiety of rapamycin in *Streptomyces hygroscopicous* by the direct cyclodeamination of l-lysine [67]. 

The *san* cluster contains the gene *san2*, encoding a protein homologous to RapL (58% identity/70% similarity) which is proposed to biosynthesize the pipecolic acid present in the molecule. In addition to rapamycin BGC, other natural product clusters also contain RapL orthologs, such as FK506/520 [68], virginiamycin S [69], tubulysin [70] or friulimicin [71].

Another structural characteristic of sandramycin is the presence of the aromatic chromophore 3-hydroxyquinaldic acid (3HQA) attached to d-Ser [39]. This chromophore is also present in other structurally related depsipeptides such as SW-163 C-G [72], thiocoraline [73], BE-22179 [74] or quinaldopeptin [75], and is the starter unit used during the assembly line of these NRPSs [76]. The biosynthesis of 3HQA, derived from l-Trp, has been described in the thiocoraline BGC [65,76]: l-Trp is first bound to TioK, a small monomodular NRPS protein formed by a didomain A–T module, which needs the structural assistance of the MbtH-like protein TioT. The attached l-Trp is then β-hydroxylated by the cytochrome P450 TioI, and the resulting β-hydroxy-l-Trp is released by the type II thioesterase TioQ. Then, the Trp-2,3-dioxygenase TioF opens the indole ring, generating *N*-formyl-β-hydroxykynurenine. Loss of this formyl moiety can be spontaneous, or other enzymes could be involved (TioL, TioM). The resulting molecule, β-hydroxykynurenine, is transformed into 3,4-dihydroxy-quinaldic acid by the aminotransferase TioG. Finally, the oxidoreductase TioH converts 3,4-dihydroxy-quinaldic acid into 3HQA. The san BGC contains genes encoding proteins homologous to TioK (*san4*), TioT (*san13*), TioI (*san5*), TioQ (*san6*), TioF (*san9*), TioG (*san8*), TioJ (*san3*) and TioH (*san7*), but no homologous to TioL or TioM were identified. This indicates that the biosynthes of the 3HQA moiety present in sandramycin occurs in the same way described for thiocoraline, but the loss of the formyl moiety of *N*-formyl-β-hydroxykynurenine may be spontaneous [76].

Similar to what has been described in thiocoraline biosynthesis [65,77], the starter unit of sandramycin would be 3HQA, that would be activated by the TioJ homolog San3. The San3-activated 3HQA may be loaded onto a fatty acid synthase (FAS) thiolation (T) domain FabC located outside of the sandramycin gene cluster, as in the case of thiocoraline [77].

Two ORFs of the *san* cluster, *san11* and *san12*, encode two NRPS containing a total of five modules (Figure 5), whose characteristics agree with the sandramycin peptide backbone (d-Ser, Pipecolic acid, l-Gly, *N*-Met-l-Gly (sarcosine) and *N*-Met-l-Val). The five modules of the NRPS contain five A domains that are predicted to activate d-Ser, l-Gly, l-Gly, l-Gly and l-Val, respectively. The first amino acid in the sandramycin peptide backbone is a d-Ser, and an epimerization domain (E) is present in the first module. The last two amino acids, l-Gly and l-Val are *N*-methylated, forming *N*-MetGly (sarcosine) and *N*-MetVal; these modifications may be performed by the *N*-methyltransferase domains (nMT) present in the last two modules. Thus, the predicted modified peptide synthesized by the NRPS, according to the collinearity rule [63], would be d-Ser, l-Gly, l-Gly, *N*-MetGly and *N*-MetVal, which agrees with the sandramycin reported backbone. Moreover, the presence of a condensation (C) domain in the first module of San11 (C1), instead of the standard loading module composed just by an A and a T domain [78], confirms that the 3HQA activated by San3 is the starter unit and is attached to the d-Ser by the C1 domain, as it has been described in similar BGCs [76]. While C1, C3, C4 and C5 condensation domains are LCL-type domains which catalyze peptide bond formation between two l-amino acids, the C2 condensation domain is a DCL-type domain, which catalyzes the condensation between a d-aminoacyl/peptidyl-T donor and a l-aminoacyl-T acceptor [79], and in the case of the sandramycin biosynthesis, this domain would catalyze the peptide bond formation between d-Ser and pipecolic acid. Finally, the thioesterase domain (TE) present in the last module would be involved in the peptide-chain release and cyclization.

**Figure 6:**
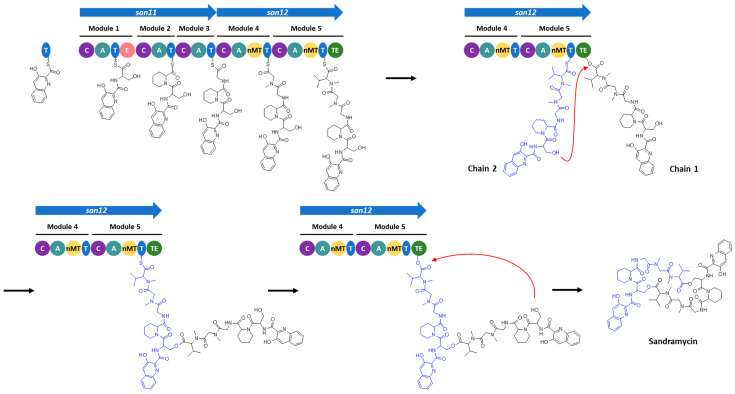
Proposed NRPS assembly line of sandramycin: two identical peptide chains are synthesized, and the T-bound PK/NRP chain is proposed to attack the thioester of the second chain tethered to the TE domain, sending it “backwards” to the ACP domain. The linear dimer is then returned to the active site of the TE domain for macrocyclization.

As sandramycin is a dimer of two identical NRP chains [39] (Figure 1), the TE domain is proposed to catalyze both the dimerization and the macrolactonization of the molecule, as it has been demonstrated in vitro with the TE domains involved in the biosynthesis of the NRP gramicidin [80] or in the polyketides conglobatin [81] and elaiophylin [82]. A “backwards transfer” mechanism was proposed for this type of biosynthesis: a T-bound PK/NRP chain is proposed to attack the thioester of an identical PK/NRP chain tethered to the downstream TE domain, sending it “backwards” to the T domain. The linear dimer is then returned to the active site of the TE domain for macrocyclization [80,83] (Figure 6). Further experiments will be needed to confirm this type of chain release mechanism.

Other genes present in the *san* BGC encode two transcriptional regulators (San10 and San15), a protein homologous to the thiocoraline resistance protein TioX (San1) [84], a HNH endonuclease (San18), two ABC transporters (San16 and San17) and an unknown protein (San14) (Figure 5, Table S4).

### 3.5. Comparative Genomic Analysis of Kribbella Strains

**Figure 7:**
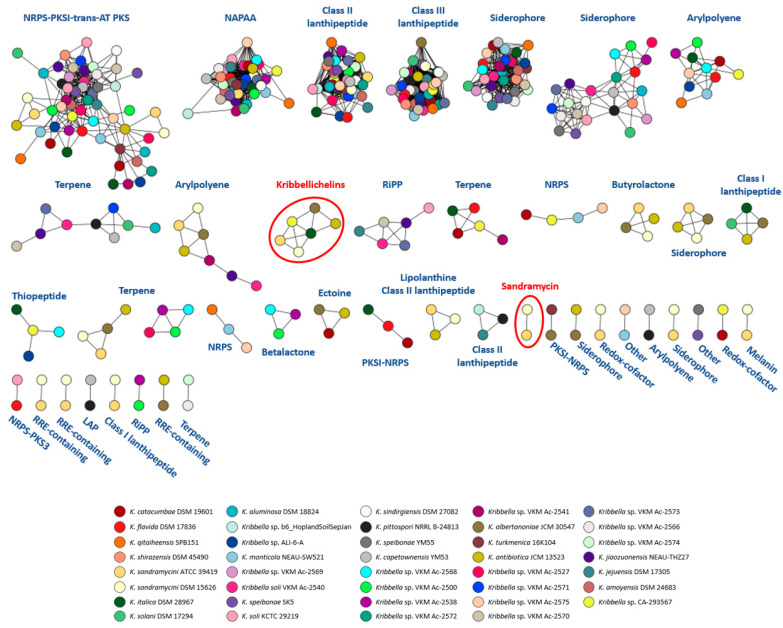
BiG-SCAPE sequence similarity networks visualized in Cytoscape. Each colour represents one *Kribbella* strain. The BGCs of sandramycin and kribbellichelins are indicated.

The publicly available genomes of 38 *Kribbella* strains (Table S1) were analyzed with antiSMASH to identify putative secondary metabolite BGCs. The 453 predicted BGCs, including those obtained from the strain CA-293567, were used as input for BiG-SCAPE/CORASON analysis to calculate the distances and to map the BGC diversity onto sequence similarity networks (Figure 7). Of the 453 BGCs analyzed, only 57 possess sequence similarities ≥70% with annotated BGCs in the MIBiG repository of antiSMASH and might produce characterized metabolites or potential analogues. Thus, nearly the 87% of the BGCs within the network might produce yet to be discovered metabolites.

The results show the presence of several groups of similar BGCs (Figure 7), which were mostly identified as NRPS-PKS-I-trans-AT PKS, NAPAA, Class II and III lanthipeptides, siderophores and RiPPs. 

**Figure 8:**
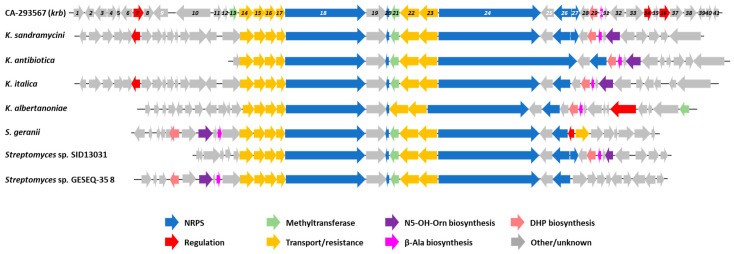
Biosynthetic gene clusters homologous to *krb* found in publicly available genomes.

The kribbellichelins BGC has also been shown to be present in the strains *K. antibiotica* JCM 13523, *K. albertanoniae* JCM 30547, *K. italica* DSM 28967 and *K. sandramycini* DSM 15626 (ATCC 39419^T^). Furthermore, a BLAST search of the *krb* biosynthetic gene cluster against the NCBI whole genome sequence database found homologous clusters in several *Streptomyces* strains (Figure 8). The organization of the cluster is very conserved among the strains, except for *Kribbella antibiotica* JCM 13523, whose NRPS2 includes an additional module incorporating β-Ala. It is worth noting that the *krb* cluster lacks genes involved in N5-OH-Orn biosynthesis, while the homologous clusters harbor a lysine/ornithine *N*-monooxygenase. However, a lysine/ornithine *N*-monooxygenase is present in the genome of the strain CA-293567, so it is probable that the kribbellichelins biosynthesis is fed with N5-OH-Orn synthesized outside the *krb* BGC.

**Figure 9:**
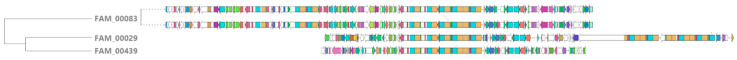
CORASON phylogenetic tree of the three Gene Cluster Families (GCF) in which sandramycin BGC has been classified. FAM_00439: sandramycin BGC from *Kribbella* sp. CA-293567; FAM_0083: sandramycin BGC from *K. sandramycini*; FAM_00029: sandramycin BGC from *Kribbella quitaiheensis* SPB151. The BGCs have been manually centered in the NRPS genes for a better visualization.

Surprisingly, the sandramycin BGC present in *Kribbella* sp. CA-293567 was not clustered with the two homologous pathways found in the *K. sandramycini* genomes (Figure 7), despite their high similarity. This may be caused by the fact that the sandramycin BGC antiSMASH prediction from *K. sandramycini* genomes includes more NRPS genes than those that are predicted in *Kribella* sp. CA-293567 in the same region. These superclusters, which are composed of subclusters with different biosynthetic features, are not properly resolved by antiSMASH, causing that BiG-SCAPE, which calculates BGC distances using a combination of the Jaccard Index of domain types, the Domain Sequence Similarity and the Adjacency Index [50], does not accurately group similar BGCs since they are assigned to different families (Figure 9), as it has been previously reported in other comparative genome analysis [85]. A BLAST search of the *san* biosynthetic gene cluster against the NCBI whole genome sequence database found a homologous cluster in the genome of *Kribbella quitaiheensis* SPB151, and neither was clustered by BiG-SCAPE (Figure 9).

## 4. Conclusions

In this work, the genome of the actinomycete strain *Kribbella* sp. CA-293567 has been sequenced, and the BGCs of sandramycin and kribbellichelins A & B have been identified. Based on the genomic predictions, a backwards transfer release mechanism is proposed for sandramycin biosynthesis, and a non-canonical post-assembly connection of two separate chains is proposed for kribbellichelins A–B biosynthesis. The number of new chain release mechanisms and new non-canonical assembly lines will grow as more genomes, especially those from minor genera of actinomycetes, are sequenced and new biosynthetic gene clusters are discovered [82]. Thus, the experimental confirmation of these proposed non-canonical mechanisms will contribute to a better understanding of PKS and NRPS biosynthesis, as well as to design new biosynthetic pathways in order to obtain new compounds.

Additionally, the genomic comparison of the publicly available *Kribbella* genomes has shown that almost 87% of the identified BGCs could putatively encode new metabolites, highlighting the biosynthetic diversity of the strains belonging to this genus. This work supports the need to continue to explore new minor genera of actinomycetes as talented sources of novel biosynthetic pathways and to study their hidden biosynthetic capabilities.

## Figures and Tables

**Table 1:** Putative biosynthetic gene clusters identified by antiSMASH in the genome of *Kribbella* sp. CA-293567.

Region	Type	From	To	Most Similar Known Cluster (MIBiG Ref)	Similarity
Region 1	RRE-containing, thiopeptide, Linear azol(in)e-containing peptide	712,243	741,688	Conglobatin (BGC0001215)	15%
Region 2	Class II lanthipeptide	896,153	919,056		
Region 3	Class III lanthipeptide	925,937	944,819	Catenulipeptin (BGC0000501)	60%
Region 4	Arylpolyene	1,300,657	1,341,691	Triacsins (BGC0001983)	6%
Region 5	Aminoglycoside/aminocyclitol cluster	2,603,585	2,624,304	Vazabitide A (BGC0001818)	6%
Region 6	NRPS-like, NRPS	2,931,152	2,996,460	Thiocoraline (BGC0000445)	34%
Region 7	Terpene	3,232,919	3,254,062	Geosmin (BGC0000661)	100%
Region 8	Siderophore	3,326,381	3,339,918		
Region 9	Class III lanthipeptide	3,364,301	3,386,850	Catenulipeptin (BGC0000501)	60%
Region 10	NRPS	3,404,482	3,470,234	A54145 (BGC0000291)	6%
Region 11	Terpene	3,492,238	3,513,224	Isorenieratene (BGC0000664)	28%
Region 12	Type I PKS, NRPS	3,555,559	3,604,623		
Region 13	NRPS	3,659,495	3,718,843	Amychelin (BGC0000300)	25%
Region 14	TransAT-PKS, NRPS, Type I PKS	5,038,833	5,103,685	Kanamycin (BGC0000703)	1%
Region 15	Type III PKS	5,114,083	5,155,129	Alkylresorcinol (BGC0000282)	100%
Region 16	RRE-containing	5,910,597	5,930,747	SCO-2138 (BGC0000595)	14%
Region 17	NAPAA	6,874,817	6,908,755		
Region 18	Redox-cofactor	7,052,415	7,074,428	Lankacidin C (BGC0001100)	20%
Region 19	Phenazine	7,585,752	7,606,243	Endophenazines A/B (BGC0001080)	33%

## Data Availability

The data presented in this study are openly available in NCBI, GenBank reference number [CP114065]. Publicly available datasets were analyzed in this study. These data can be found in NCBI, GenBank reference numbers [CP001736.1, AQUZ00000000.1, MTQN00000000.1, QFXK00000000.1, SGXJ00000000.1, SHKR00000000.1, SJJY00000000.1, SJJZ00000000.1, SJKC00000000.1, SJKA00000000.1, SJKB00000000.1, SJKD00000000.1, SLVW00000000.1, SLWN00000000.1, SLWM00000000.1, SLVF00000000.1, SLWR00000000.1, SMKA00000000.1, SMKR00000000.1, SMKX00000000.1, SNWQ00000000.1, SNWS00000000.1, SOCE00000000.1, SODP00000000.1, SODF00000000.1, SODT00000000.1, SODU00000000.1, SZPZ00000000.1, VFMM00000000.1, VIVK00000000.1, JAASRO010000001.1, JABJRC000000000.1, JACHMY000000000.1, JACHNF000000000.1, JACHKF010000001.1, CP043661.1, JAGINT000000000.1, JAJKIE000000000.1].

## Supplementary Materials

The following supporting information can be downloaded at: https://www.mdpi.com/article/10.3390/microorganisms11020265/s1, Table S1: Publicly available genomes from *Kribbella* strains used in this study; Table S2: *Kribbella* sp. CA-293567 genome statistics; Table S3: ORFs present in the kribbellichelins A-B BGC; Table S4: ORFs present in the sandramycin BGC from *Kribbella* sp. CA-293567.
