# Supplementary material for: Complete Genome Sequence Analysis of Kribbella sp. CA-293567 and Identification of the Kribbellichelins A & B and Sandramycin Biosynthetic Gene Clusters
Source: Microorganisms. 2023 Jan 19;11(2):265. doi: 10.3390/microorganisms11020265 (PMC9962454; doi:10.3390/microorganisms11020265)
Supplement: Supplementary file 1 [file microorganisms-11-00265-s001.zip › microorganisms-2142687-supplementary.pdf]

Supplementary Information

Complete Genome Sequence Analysis of *Kribbella* sp. CA-293567 and Identification of the Kribbellichelins A & B and Sandramycin Biosynthetic Gene Clusters

Marina Sánchez-Hidalgo*, María Jesús García, Ignacio González, Daniel Oves-Costales, Olga Genilloud

Fundación MEDINA, Avenida del Conocimiento 34, PTS Health Sciences Technology Park, 18016 Granada, Spain
* Correspondence: marina.sanchez@medinaandalucia.es (M.S-H.), +34958993965

Table S1. Publicly available genomes from *Kribbella* strains used in this study. The genome accession codes and number of antiSMASH predicted regions are indicated.

Strain	GenBank accession	Assembly code	WGS accession	Assembly Level	Number of contigs	Size	GC content	Number of antiSMASH predicted regions
Kribbella flavida DSM 17836	GCA_000024345.1	ASM2434v1	CP001736.1	Complete	1	7,579,488	70.5	12
Kribbella catacumbae DSM 19601	GCA_000372465.1	ASM37246v1	AQUZ00000000.1	Contig	152	9,627,810	67.5	17
Kribbella sp. ALI-6-A	GCA_001984195.1	ASM198419v1	MTQN00000000.1	Contig	12	8,101,914	69.5	12
Kribbella monticola NEAU-SW521	GCA_003261635.1	ASM326163v1	QFXK00000000.1	Contig	92	8,028,932	67.8	12
Kribbella sp. VKM Ac-2569	GCA_004216905.1	ASM421690v1	SGXJ00000000.1	Contig	13	8,427,108	68	7
Kribbella soli VKM Ac-2540	GCA_004217145.1	ASM421714v1	SHKR00000000.1	Contig	22	8,637,800	67.5	9
Kribbella speibonae SK5	GCA_004331335.1	ASM433133v1	SJJY00000000.1	Contig	21	8,874,969	68.6	9
Kribbella soli KCTC 29219	GCA_004331345.1	ASM433134v1	SJJZ00000000.1	Contig	10	8,785,001	68	9
Kribbella speibonae YM55	GCA_004331375.1	ASM433137v1	SJKC00000000.1	Contig	17	9,285,392	68.6	9
Kribbella sindirgiensis DSM 27082	GCA_004331435.1	ASM433143v1	SJKA00000000.1	Contig	54	8,891,727	68.5	8
Kribbella pittospori NRRL B-24813	GCA_004331465.1	ASM433146v1	SJKB00000000.1	Contig	70	9,491,812	68.1	12
Kribbella capetownensis YM53	GCA_004331485.1	ASM433148v1	SJKD00000000.1	Contig	31	8,831,527	68	9
Kribbella sp. VKM Ac-2500	GCA_004340265.1	ASM434026v1	SLVW00000000.1	Contig	82	9,277,683	68	9
Kribbella sp. VKM Ac-2572	GCA_004342025.1	ASM434202v1	SLWN00000000.1	Contig	45	9,146,758	68	10
Kribbella sp. VKM Ac-2538	GCA_004342085.1	ASM434208v1	SLWM00000000.1	Contig	73	9,192,871	68	8
Kribbella sp. VKM Ac-2568	GCA_004345365.1	ASM434536v1	SLVF00000000.1	Contig	41	8,875,416	68	10
Kribbella sp. VKM Ac-2541	GCA_004345665.1	ASM434566v1	SLWR00000000.1	Contig	33	8,102,898	67.5	13
Kribbella albertanoniae JCM 30547	GCA_004348455.1	ASM434845v1	SMKA00000000.1	Contig	593	9,727,339	67.4	25
Kribbella turkmenica 16K104	GCA_004348725.1	ASM434872v1	SMKR00000000.1	Contig	351	7,447,142	69.4	10
Kribbella antibiotica JCM 13523	GCA_004349055.1	ASM434905v1	SMKX00000000.1	Contig	348	9,279,240	67	33
Kribbella sp. VKM Ac-2527	GCA_004361855.1	ASM436185v1	SNWQ00000000.1	Contig	98	9,457,695	67.5	12
Kribbella sp. VKM Ac-2571	GCA_004362255.1	ASM436225v1	SNWS00000000.1	Contig	50	8,494,871	68	8
Kribbella sp. VKM Ac-2575	GCA_004365175	ASM436517v1	SOCE00000000.1	Contig	9	8,032,448	68	13
Kribbella sp. VKM Ac-2573	GCA_004365355.1	ASM436535v1	SODP00000000.1	Contig	15	8,365,383	68	8
Kribbella sp. VKM Ac-2570	GCA_004365875.1	ASM436587v1	SODF00000000.1	Contig	12	8,322,687	67.5	9
Kribbella sp. VKM Ac-2566	GCA_004366005.1	ASM436600v1	SODT00000000.1	Contig	25	8,670,423	68	8
Kribbella sp. VKMAc-2574	GCA_004366075.1	ASM436607v1	SODU00000000.1	Contig	14	8,387,673	68	7
Kribbella jiaozuonensis NEAU-THZ27	GCA_005233875.1	ASM523387v1	SZPZ00000000.1	Contig	12	8,659,701	68	10
Kribbella jejuensis DSM 17305	GCA_006715085.1	ASM671508v1	VFMM00000000.1	Contig	4	7,505,702	68.5	9
Kribbella amoyensis DSM 24683	GCA_007828865.1	ASM782886v1	VIVK00000000.1	Contig	8	8,120,218	70	9
Kribbella shirazensis DSM 45490	GCA_011761605.1	ASM1176160v1	JAASRO010000001.1	Contig	1	8,596,726	69	7
Kribbella sandramycini ATCC 39419	GCA_013131805.1	ASM1313180v1	JABJRC000000000.1	Contig	34	8,105,579	69.1	19
Kribbella italica DSM 28967	GCA_014205135.1	ASM1420513v1	JACHMY000000000.1	Contig	1	8,885,108	69.5	14
Kribbella solani DSM 17294	GCA_014205295.1	ASM1420529v1	JACHNF000000000.1	Contig	2	7,851,691	68	11
Kribbella sandramycini DSM 15626	GCA_014207795.1	ASM1420779v1	JACHKF010000001.1	Contig	1	8,120,153	69	20
Kribbella qitaiheensis SPB151	GCA_014217565.1	ASM1421756v1	CP043661.1	Complete	1	8,156,807	67	12
Kribbella aluminosa DSM 18824	GCA_017876295.1	ASM1787629v1	JAGINT000000000.1	Contig	2	8,936,317	68.5	11
Kribbellla sp. b6_HoplandSoilSeptJan	GCA_021154635.1	ASM2115463v1	JAJKIE000000000.1	Contig	425	4,085,102	68	4
Kribbella sp. CA-293567				Complete	1	7,611,196	68.6	19

Table S2. *Kribbella* sp. CA-293567 genome statistics.

Characteristic	Value	% of Total
Genome size (bp)	7,611,196	
DNA G+C content (bp)		68.6
Number of replicons	1	
Extrachromosomal elements	0	
Total genes	7,057	100
Protein-coding genes	6982	98.93
Genes with function prediction	3197	45.3

Table S3. ORFs present in the kribbellichelins A-B BGC.

ORF	Length of encoded protein (AA)	Closest BLAST homolog [Strain] Ref	Identity (%)	Similarity (%)
krb1	352	Hypothetical protein [*Kribbella catacumbae*] WP_020384918.1	71	79
krb2	275	Undecaprenyl-diphosphate phosphatase [*Streptomyces* sp. SID13031] WP_164593903.1	95	98
krb3	356	LLM class F420-dependent oxidoreductase [*Kribbella catacumbae*] WP_026162457.1	95	97
krb4	313	Aldo/keto reductase [*Kribbella* sp. VKM Ac-2568] WP_132297909.1	99	100
krb5	61	DUF5703 family protein [*Kribbella qitaiheensis*] WP_185445878.1	100	100
krb6	405	Major facilitator superfamily MFS_1 [*Kribbella flavida* DSM 17836] ADB32869.1	76	85
krb7	295	LysR family transcriptional regulator [*Kribbella* sp. VKM Ac-2568] WP_132297890.1	83	89
krb8	252	SDR family oxidoreductase [*Acrocarpospora macrocephala*] WP_155361669.1	69	82
krb9	443	M20/M25/M40 family metallo-hydrolase [*Streptomyces* sp. SID13031] WP_164593881.1	95	97
krb10	1051	S8 family serine peptidase [*Streptomyces* sp. SID13031] WP_164593874.1	93	96
krb11	260	Hypothetical protein [*Amycolatopsis keratiniphila*] WP_043848172.1	52	63
krb12	133	Hypothetical protein [*Amycolatopsis alba*] WP_020634912.1	60	76
krb13	263	Class I SAM-dependent methyltransferase [*Kribbella* sp. VKM Ac-2575] WP_133979490.1	92	94
krb14	417	ABC transporter substrate-binding protein [*Kribbella speibonae*] WP_131462990.1	83	91
krb15	348	Iron chelate uptake ABC transporter family permease subunit [*Kribbella antibiotica*] WP_132168665.1	93	97
krb16	369	Iron chelate uptake ABC transporter family permease subunit [*Kribbella speibonae*] WP_202875736.1	90	93
krb17	294	ABC transporter ATP-binding protein [*Streptomyces* sp. SID13031] WP_164596070.1	95	97
krb18	2463	Non-ribosomal peptide synthetase [*Streptomyces* sp. SID13031] WP_164596008.1	84	89
krb19	616	Siderophore-interacting protein [*Streptomyces* sp. SID13031] NEA32353.1	88	93
krb20	83	Phosphopantetheine-binding protein [*Streptomyces* sp. SID13031] WP_164596006.1	93	96
krb21	285	Class I SAM-dependent methyltransferase [*Streptomyces* sp. SID13031] WP_164596005.1	95	97
krb22	581	ABC transporter ATP-binding protein [*Streptomyces* sp. SID13031] NEA32350.1	92	95
krb23	556	ABC transporter ATP-binding protein/permease [*Kribbella italica*] WP_202893055.1	90	93
krb24	3107	Amino acid adenylation domain-containing protein [*Streptomyces* sp. SID13031] WP_239062464.1	85	90
krb25	406	FAD-dependent monooxygenase [*Streptomyces* sp. SID13031] WP_164596002.1	92	96
krb26	541	AMP-binding protein [*Kribbella albertanoniae*] WP_132402990.1	96	98
krb27	232	4'-phosphopantetheinyl transferase superfamily protein [*Streptomyces* sp. SID13031] WP_164596000.1	89	92
krb28	273	Alpha/beta hydrolase [*Kribbella albertanoniae*] WP_132402993.1	89	92
krb29	248	4-hydroxy-tetrahydrodipicolinate reductase [*Streptomyces* sp. SID13031] WP_164595998.1	90	95
krb30	137	Aspartate 1-decarboxylase [*Streptomyces* sp. SID13031] WP_164595997.1	97	99
krb31	80	MbtH family NRPS accessory protein [*Kribbella albertanoniae*] WP_132403002.1	97	98
krb32	490	FAD-dependent tricarballylate dehydrogenase TcuA [*Kribbella albertanoniae*] WP_132403005.1	94	96
krb33	546	Alpha/beta hydrolase [*Kribbella flavida*] WP_041289211.1	88	93
krb34	242	MerR family transcriptional regulator [*Kribbella albertanoniae*] WP_132405175.1	83	89
krb35	156	Nuclear transport factor 2 family protein [*Streptomyces phaeochromogenes*] WP_055611607.1	78	88
krb36	206	TetR/AcrR family transcriptional regulator [*Kibdelosporangium persicum*] WP_173131860.1	78	88
krb37	400	Pimeloyl-ACP methyl ester carboxylesterase [*Kribbella* sp. VKM Ac-2571] TDO68387.1	91	95
krb38	431	Epoxide hydrolase [*Kribbella* sp. VKM Ac-2571] WP_133782690.1	89	93
krb39	220	DUF998 domain-containing protein [*Kribbella turkmenica*] WP_132321484.1	77	87
krb40	112	Hypothetical protein [*Kribbella flavida*] WP_012919172.1	81	91
krb41	181	Hypothetical protein [*Kribbella amoyensis*] WP_145806086.1	72	79

Table S4. ORFs present in the sandramycin BGC from *Kribbella* sp. CA-293567. A comparison with the ORFs present in the *sdm* and *tio* BGCs is shown.

ORF	Length of encoded protein (AA)	Closest BLAST homolog [Strain] Ref	Identity (%)	Similarity (%)	*K. sandramycini* homologous gene	Identity (%)	Similarity (%)	Thiocoralline homologous gene	Identity (%)	Similarity (%)
san1	144	VOC family protein [*Kribbella qitaiheensis*] WP_185445859.1	81	92	***sdm1***	73.4	81.1	***tioX***	56.7	70.1
san2	355	Ornithine cyclodeaminase family protein [*Kribbella qitaiheensis*] WP_185445860.1	90	94	***sdm2***	84.6	92.4			
san3	526	AMP-binding protein [*Kribbella qitaiheensis*] WP_185445861.1	84	91	***sdm3***	83.7	89.5	***tioJ***	63.5	78.2
san4	582	Non-ribosomal peptide synthetase [*Kribbella qitaiheensis*] WP_185445862.1	83	90	***sdm4***	81.3	88	***tioK***	61.5	74.4
san5	398	Cytochrome P450 [*Amycolatopsis* sp. MtRt-6] WP_206792488.1	88	94	***sdm5***	85.7	91.7	***tioI***	65.2	77.4
san6	257	Thioesterase [*Kribbella qitaiheensis*] QNE17028.1	77	86	***sdm6***	74.7	79.4	***tioQ***	48.6	62.4
san7	248	NAD(P)-dependent dehydrogenase [*Kribbella sandramycini*] MBB6569689.1	78	89	***sdm7***	78	89	***tioH***	58.7	71.6
san8	409	Pyridoxal phosphate-dependent aminotransferase [*Amycolatopsis bullii*] WP_191307014.1	87	92	***sdm8***	86.6	91.4	***tioG***	61.8	74.5
san9	239	Tryptophan 2,3-dioxygenase family protein [*Kribbella qitaiheensis*] WP_185445866.1	87	93	***sdm9***	86.4	95	***tioF***	58.8	71.6
san10	168	DNA-binding response OmpR family regulator [*Kribbella sandramycini*] MBB6569686.1	90	94	***sdm10***	90	94			
san11	3635	Amino acid adenylation domain-containing protein [*Kribbella qitaiheensis*] QNE17032.1	76	85	***sdm11***	74.7	83.8			
san12	3090	Non-ribosomal peptide synthetase [*Kribbella qitaiheensis*] WP_185445868.1	77	85	***sdm12***	73.1	82.9			
san13	73	MbtH family NRPS accessory protein [*Amycolatopsis* sp. MtRt-6] WP_206792493.1	85	91	***sdm13***	68.5	82.2	***tioT***	70.4	84.5
san14	145	Hypothetical protein [*Rhodococcus marinonascens*] WP_245813747.1	65	76	***sdm14***	65.8	76			
san15	233	TetR familytranscriptional regulator [*Streptomyces* sp. SID13031] WP_164593841.1	83	88	-	-	-			
san16	321	ABC transporter ATP-binding protein [*Kribbella qitaiheensis*] WP_185445870.1	87	92	***sdm15***	78.3	88.2			
san17	283	ABC transporter permease [*Kribbella qitaiheensis*] WP_185445871.1	83	88	***sdm16***	79.7	88.6			
san18	228	HNH endonuclease family protein [*Streptomyces* sp. SID13031] WP_164593685.1	90	94	***sdm17***	71.5	78.5			
san19	475	Aminopeptidase P family protein [*Kribbella catacumbae*] WP_020390480.1	81	86	-	-	-			
